# CD73 mitigates ZEB1 expression in papillary thyroid carcinoma

**DOI:** 10.1186/s12964-024-01522-z

**Published:** 2024-02-22

**Authors:** Samlai Vedovatto, Fernanda Dittrich Oliveira, Luiza Cherobini Pereira, Thamiris Becker Scheffel, Liziane Raquel Beckenkamp, Ana Paula Santin Bertoni, Márcia Rosângela Wink, Guido Lenz

**Affiliations:** 1 Department of Biophysics, Federal University of Rio Grande do Sul, Av. Bento Gonçalves, 9500, Prédio 43431, sala 107, UFRGS, Porto Alegre, RS Brazil; 2 Brain Institute of Rio Grande do Sul, Pontifical Catholic University of Rio Grande do Sul, Porto Alegre, RS Brazil; 3 Department of Basics Health Sciences and Laboratory of Cell Biology, Federal University of Health Sciences of Porto Alegre, Porto Alegre, RS Brazil

**Keywords:** CD73, ZEB1, Papillary thyroid carcinoma, Epithelial-mesenchymal plasticity, Adenosinergic signaling

## Abstract

**Background:**

ZEB1, a core transcription factor involved in epithelial-mesenchymal transition (EMT), is associated with aggressive cancer cell behavior, treatment resistance, and poor prognosis across various tumor types. Similarly, the expression and activity of CD73, an ectonucleotidase implicated in adenosine generation, is an important marker of tumor malignancy. Growing evidence suggests that EMT and the adenosinergic pathway are intricately linked and play a pivotal role in cancer development. Therefore, this study focuses on exploring the correlations between CD73 and ZEB1, considering their impact on tumor progression.

**Methods:**

We employed CRISPR/Cas9 technology to silence CD73 expression in cell lines derived from papillary thyroid carcinoma. These same cells underwent lentiviral transduction of a reporter of ZEB1 non-coding RNA regulation. We conducted studies on cell migration using scratch assays and analyses of cellular speed and polarity. Additionally, we examined ZEB1 reporter expression through flow cytometry and immunocytochemistry, complemented by Western blot analysis for protein quantification. For further insights, we applied gene signatures representing different EMT states in an RNA-seq expression analysis of papillary thyroid carcinoma samples from The Cancer Genome Atlas.

**Results:**

Silencing CD73 expression led to a reduction in ZEB1 non-coding RNA regulation reporter expression in a papillary thyroid carcinoma-derived cell line. Additionally, it also mitigated ZEB1 protein expression. Moreover, the expression of CD73 and ZEB1 was correlated with alterations in cell morphology characteristics crucial for cell migration, promoting an increase in cell polarity index and cell migration speed. RNA-seq analysis revealed higher expression of *NT5E* (CD73) in samples with BRAF mutations, accompanied by a prevalence of partial-EMT/hybrid state signature expression.

**Conclusions:**

Collectively, our findings suggest an association between CD73 expression and/or activity and the post-transcriptional regulation of ZEB1 by non-coding RNA, indicating a reduction in its absence. Further investigations are warranted to elucidate the relationship between CD73 and ZEB1, with the potential for targeting them as therapeutic alternatives for cancer treatment in the near future.

**Supplementary Information:**

The online version contains supplementary material available at 10.1186/s12964-024-01522-z.

## Introduction

Epithelial-mesenchymal transition (EMT) is a fundamental biological process in which cells undergo a transformation from an epithelial to a mesenchymal phenotype. EMT plays a pivotal role in embryonic development, tissue repair, and cancer metastasis [1, 2]. Zinc finger E-box binding homeobox 1 (ZEB1), a master transcription factor in the EMT program, is intricately linked to aggressive behavior, metastasis, treatment resistance, and poor prognosis across diverse tumor types [3–5]. Several signaling pathways converge to regulate ZEB1 activity, placing particular emphasis on the ZEB1/miR-200 double-negative feedback loop. This loop encompasses eight miR-200 family binding sites that actively repress EMT, thereby influencing ZEB1’s involvement in tumor development [6, 7].

Adenosinergic signaling involves the production and action of adenosine, a molecule that modulates a wide array of cellular functions including inflammation, immunity, angiogenesis, and apoptosis [8]. Adenosine is generated from the hydrolysis of adenosine monophosphate (AMP) through the catalytic activity of the CD73 enzyme, encoded by the *NT5E* gene. CD73’s actions can have immunosuppressive effects within the tumor microenvironment [8]. In the context of papillary thyroid carcinoma (PTC), which constitutes approximately 90% of all thyroid carcinoma cases, CD73 emerges as a critical contributor. Its overexpression is intricately associated with tumor progression, cell migration, proliferation, and particularly with cells situated at the invasive front of PTC [9–13]. Despite a 10-year survival rate of approximately 90%, PTC presents ongoing challenges with recurrent cases, requiring surgical procedures and treatment involving radioactive iodine ablation [14, 15].

Evidence underscores the intricate interplay between EMT and adenosinergic signaling, highlighting its pivotal role in cancer initiation and progression [16]. Beyond CD73’s known immunosuppressive effects within the tumor microenvironment through adenosine, CD73 may also contribute to the early stages of tumor progression by facilitating EMT [17]. CD73 suppression has been shown to increase E-cadherin expression while decreasing vimentin expression, favoring a more epithelial phenotype [17]. Given this potential connection, conducting a comprehensive examination of the interaction between adenosinergic signaling and EMT becomes imperative. This study is dedicated to investigating the impact of CD73 on ZEB1, aiming to elucidate the intricate connections between them and understand their implications in the progression of cancer.

## Materials and methods

### Cell culture

Human thyroid cancer cell lines (TPC and BCPAP), which had undergone CRISPR/Cas9-mediated deletion of CD73, were generously provided by the Cell Biology Laboratory at the Federal University of Health Sciences of Porto Alegre (UFCSPA). The cell lines were cultured in high-glucose Dulbecco’s modified Eagle’s medium (DMEM; Gibco, Massachusetts, EUA) supplemented with 10% fetal bovine serum (FBS; Gibco, Massachusetts, EUA), 1% ampicillin/streptomycin, and 0.1% amphotericin. The cells were maintained at 37 °C in a 5% CO2 atmosphere.

### CRISPR/Cas9

The CRISPR/Cas9 deletion of CD73 in the TPC and BCPAP cell lines was executed by the Cell Biology Laboratory at UFCSPA. The procedure employed a plasmid sequence provided by The Cancer Research Institute of Montreal, containing a guide RNA (gRNA) inserted into a pX330-U6-Chimeric_BBCBh-hSpCas9 vector, designed to edit the human *NT5E* gene and silence CD73 expression [18, 19]. The corresponding sequence is L3-GCAGCACGTTGGGTTCGGCG. Initially, 10^4^ cells were seeded into a 6-well plate and cultured for 3 days. Subsequently, they were transfected with 1 mg of the plasmid using Lipofectamine LTX (ThermoFisher Scientific Inc., Rockford, USA), following the manufacturer’s instructions.

### Ectonucleotidase assay / enzymatic activity

For the ectonucleotidase assay, 10^3^ cells were seeded in 24-well plates and grown to 90–95% confluency [13]. Afterward, cells were washed three times with an incubation medium containing 2 mM MgCl_2_, 120 mM NaCl, 5 mM KCl, 10 mM glucose, and 20 mM HEPES - pH 7.4 (Sigma–Aldrich, St. Louis, U.S.A.). The reaction was initiated by adding 200 μL of incubation medium containing 2 mM of AMP, followed by incubation at 37 °C for different durations (15, 30, and 60 minutes). After the specified incubation times, 150 μL of the incubation medium with nucleotides was transferred into microtubes containing 150 μL of 10% w/v trichloroacetic acid. The release of inorganic phosphate (Pi) was quantified using the Malachite Green method with measurements taken at 630 nm using a SpectraMax Microplate Reader (Molecular Devices Corporation, USA). Normalization was performed based on protein measurements using the Bradford method at 595 nm.

### Lentiviral transduction of ZEB1

Lentivirus production involved co-transfecting HEK cells with three plasmids: psPAX2 (packaging plasmid - Addgene_12260), pMD2.G (VSV-G envelope expressing plasmid - Addgene_12259), and FUGW-d2GFP-ZEB1 3’UTR (Addgene_79601). Transfection was carried out using Lipofectamine™ 3000 reagent (Invitrogen/Thermo Fisher Scientific) as per the manufacturer’s instructions. Following supernatant collection and filtration (0.45 μm membrane), the target cells were transduced with the ZEB1 non-coding RNA regulation reporter in the presence of polybrene (8 mg/mL). Transduced cells were then centrifuged (45 minutes at 700 g, 25 °C) and incubated overnight. Subsequently, cell culture media were replaced, and fluorescence expression was monitored before initiating puromycin (8 μg/mL) selection for 48 hours.

### Scratch wound assay

The scratch wound assay was conducted by gently dragging 200 μL micropipette tips across culture plates (6-well) with a cell confluence of 100%. After scratching, the wells were washed twice with PBS to remove detached cells, and the remaining cells were incubated in a standard medium with 1% fetal bovine serum (FBS; Gibco, Massachusetts, EUA). Each experimental group was analyzed in triplicate. Images were captured using the IncuCyte Live-Cell imaging system (S3/SX1 G/R Optical Module) at 0 hours and 12 hours post-scratching. Image analysis was performed using ImageJ software (National Institute of Health, USA).

### Immunocytochemistry

TPC and BCPAP cells underwent immunocytochemistry using Anti-ZEB1 (NBP1-05987) following the manufacturer’s instructions. Briefly, cells were washed with PBS following DMEM removal, fixed with 4% PFA for 20 minutes at room temperature, and then washed thrice with PBS. A blocking solution (300 mg BSA, 30 μL Triton X-100, 10 mL PBS 1x) was used for incubation for 1 hour. Primary antibody was applied to coverslips and incubated overnight at 4 °C, followed by three PBS washes. Subsequently, a secondary antibody was added and incubated for 1 hour. Finally, DAPI (300 nM) staining was performed with a 20-minute incubation. Images were acquired using the EVOS FLoid Imaging System (ThermoFisher Scientific).

### Western blotting

BCPAP and TPC cells (0.6 × 10^6^) were washed three times with cold PBS 1x, followed by lysis in 60 μL of lysis buffer (50 mM Tris-HCl pH 6.8, 2 mM EDTA, 4% SDS), and subsequent heating at 75 °C for 5 min. The total protein concentration was determined using the Pierce BCA Protein Assay Kit (Thermo Scientific). The whole-cell lysate (30 μg) was loaded into each lane, resolved in a 12% SDS-PAGE gel, and transferred to PVDF membranes. Rabbit anti-ZEB1 (1:2000, Novus Biologicals #NBP1-05987) served as the primary antibody, and a peroxidase-conjugated anti-rabbit (1:1000, Cell Signaling #7074) was employed as the secondary antibody.

### Image analysis and quantification

Images obtained from the IncuCyte Live-Cell imaging system (S3/SX1 G/R Optical Module) were analyzed using ImageJ software (National Institute of Health, USA). For cell polarity index calculations, the length of the primary migration axis was divided by the length of the perpendicular axis, which intersects the cell nucleus. Migration speed was determined using the Manual Tracking plugin, and fluorescence of the ZEB1 reporter was quantified as the integrated density mean normalized by area. Additionally, cell cytoplasmic and nuclear quantifications were validated using the Python-based ForNMA code developed by Luiza Cherobini Pereira (unpublished).

### The Cancer genome atlas (TCGA) data analysis

RNA-seq expression data of PTC patients were retrieved from the TCGA PanCancer Atlas dataset through the cBioPortal for Cancer Genomics [20, 21]. To compare gene and signature expression among patient groups, data normality was assessed using the Shapiro-Wilk normality test. Since the data did not meet the normality assumption, nonparametric statistical tests, namely the Mann-Whitney U test or Kruskal-Wallis test followed by Dunn’s multiple comparison test, were selected. The gene signatures representing different EMT states were adapted from Pastushenko and Blainpain (2019) and included the following categories: Epithelial (*KRT5, KRT14, DSG2, ESRP1, ESRP2, TP63, KLF4, OVOL1, GRHL1, GRHL2, GRHL3),* Hybrid *(KRT5, KRT14, VIM, PDGFRB, FAP, CDH2, TP63, GRHL1, GRHL2, GRHL3, ZEB1, ZEB2, TWIST1, TWIST2, SNAI1),* and Mesenchymal *(VIM, ASPN, CDH2, FAP, MMP19, LOX, PRRX1, ZEB1, ZEB2, TWIST1, TWIST2, SNAI1*) [22]. Patient grouping based on EMT profiles, utilizing the Epithelial and Mesenchymal signatures, was carried out following the approach by Pereira et al. (2018) [23].

### Statistical analysis

Statistical analyses were conducted using GraphPad Prism version 8.0.2 for Windows (GraphPad Software, San Diego, California, USA). Student’s t-test and correlation coefficient calculations were employed, with statistical significance considered when *p* < 0.05. Clinical features derived from TCGA data were subjected to analysis using SPSS 19.0 software. Descriptive and univariate analyses of EMT states among clinical features were performed using the chi-square test or Fisher’s exact test for dichotomous variables. Multivariate analysis employed Generalized Linear Models (GLM) with a gamma distribution and a logarithmic link function to investigate relationships between EMT states and clinical features. All analyses were two-sided, and *p* values < 0.05 were deemed statistically significant.

## Results

### CD73 deletion results in decreased expression of the ZEB1 non-coding RNA regulation reporter in the BCPAP cell line

**Fig. 1:**
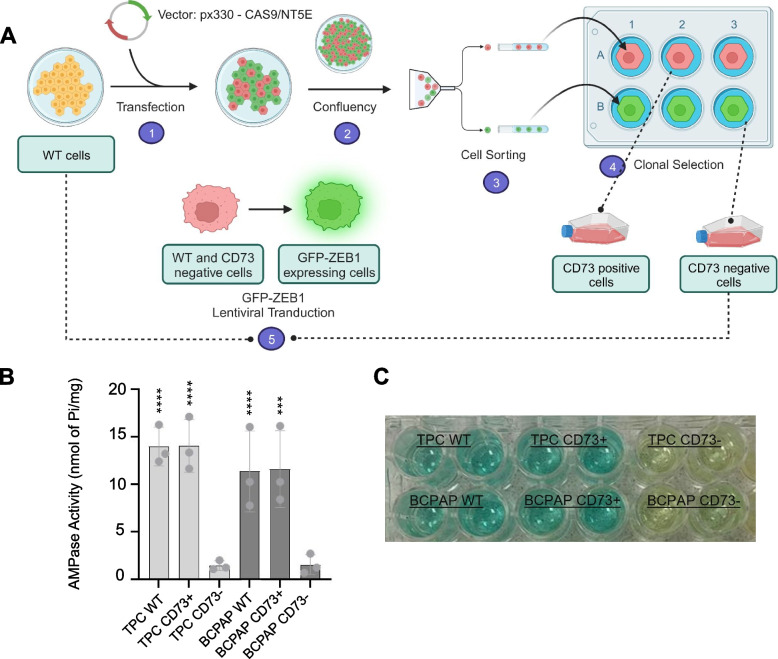
Schematic representation of the editing process for CD73 deletion and transduction of ZEB1 regulation reporter in PTC cell lines (**A**). Measurement of AMPase activity (nmol PI/mg of protein) in TPC and BCPAP cell lines after the editing process (**B**). Enzymatic activity curve of TPC and BCPAP cells (**C**)

**Fig. 2:**
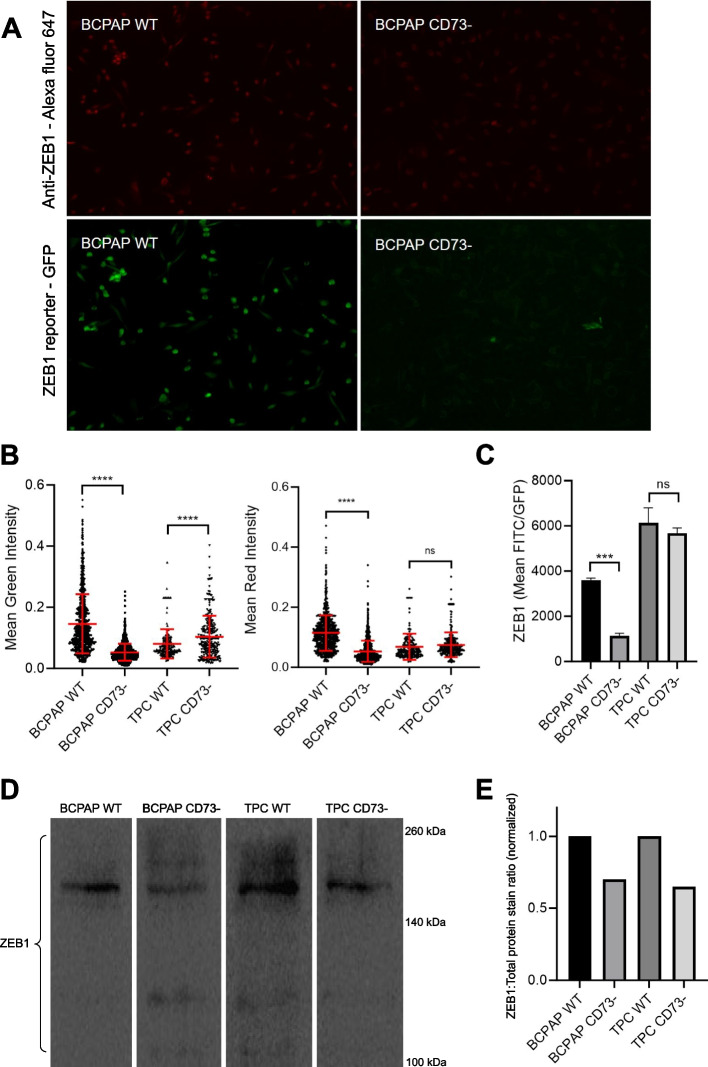
ZEB1 reporter expression and Anti-ZEB1 immunocytochemistry. **A** Fluorescence representation of mean ZEB1 reporter intensity and mean ZEB1 ICC in BCPAP WT and BCPAP CD73- cells. **B** Violin plots illustrating cell distribution based on mean ZEB1 reporter intensity and mean ZEB1 ICC. **C** Bar plots of ZEB1 reporter expression from flow cytometry. Expression of ZEB1’s non-coding RNA regulation reporter (GFP/FITC) is presented based on mean values of the triplicate. *** = *p* ≤ 0.001; **** = *p* ≤ 0.0001; ns = not significant. **D** Representative Western blot showing the expression levels of ZEB1 protein in lysates of cell lines derived from PTC. **E** Densitometric analysis of the Western blot. Band intensities were quantified using ImageJ software. The expression levels of ZEB1 protein were normalized to the total protein stain from respective lanes

Wildtype cells were subjected to CD73 silencing along with fluorescent ZEB1 expression (Fig. 1A). CD73 editing by CRISPR/Cas9 was confirmed by enzymatic activity measurement. As expected, there was a decrease in AMPase hydrolysis rate in CD73- cells (Fig. 1B, C). BCPAP and TPC cells that underwent editing for CD73 silencing by CRISPR-Cas9 and lentiviral transduction for expression of the ZEB1 non-coding RNA regulation reporter were analyzed to assess differences in ZEB1 reporter expression. CD73 deletion reduced ZEB1 immunocytochemistry (ICC) signal, confirming the reduction observed with the live-cell reporter in BCPAP cells (Fig. 2A, B), whereas in TPC cells deletion of CD73 did not affect the expression of ZEB1 (Fig. 2B). Flow cytometry confirmed the reduction of ZEB1 expression with the deletion of CD73 in BCPAP cells, and Western blot analysis revealed an approximate 30% decrease in ZEB1 protein levels in CD73- cells, observed in both TPC and BCPAP cell lines (Fig. 2C-E).

### CD73 deletion reduces cell polarity in the BCPAP cell line

**Fig. 3:**
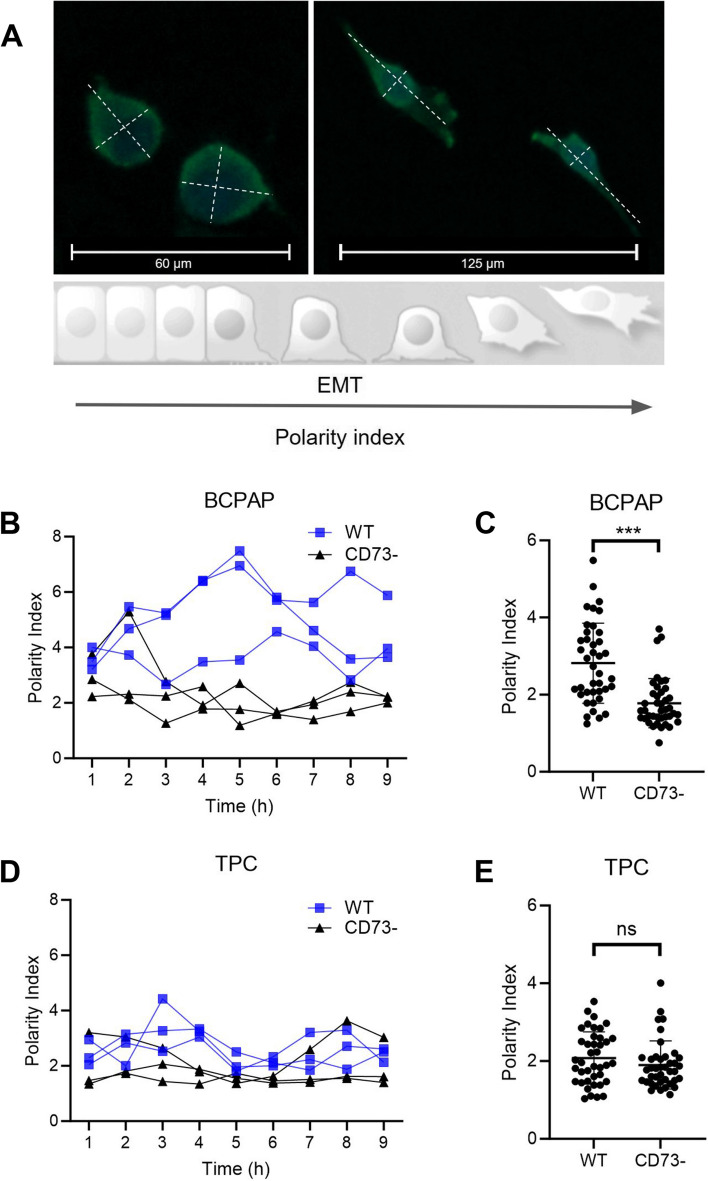
Polarity index of PTC cell lines and its relation to EMT morphological phenotypes. **A** Schematic representation of cell morphology from low to high polarity index, respectively: epithelial (right) and mesenchymal (left). Merged images of DAPI and phalloidin staining of BCPAP cells. **B** Polarity index of BCPAP representative individual cells over time (hours). **C** Scatter plot comparing individual cells from BCPAP WT and BCPAP CD73- groups. **D** Polarity index of TPC representative individual cells over time (hours). **E** Scatter plot comparing individual cells from TPC WT and TPC CD73- groups. *** = *p* ≤ 0.001; ns = not significant

The assessment of cell polarity index serves as an indicator of epithelial (round shape) or mesenchymal (elongated shape) phenotypes, which is a relevant metric in the context of correlating morphological changes with EMT (Fig. 3A). BCPAP CD73- cells displayed significantly lower polarity compared to BCPAP WT cells (Fig. 3B, C). This observation suggests a specific contribution of CD73 to cytoskeletal configuration in this cell line. Conversely, such differences were not observed in the TPC cell line (*p* < 0.0001) (Fig. 3D, E).

### CD73 deletion reduces cell migration in the BCPAP cell line

**Fig. 4:**
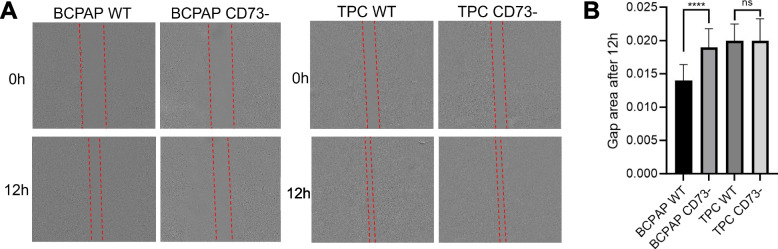
Scratch wound assay of BCPAP and TPC cells at 0 h and 12 h. **A** Scratch wound assay representation (dotted lines). **B** Graphical representation of the gap area after 12 h. **** = *p* ≤ 0.0001; ns = not significant

TPC WT and TPC CD73- cells demonstrated similar gap closure times. In contrast, BCPAP CD73- cells exhibited a longer time to close the gap area compared to WT cells in the BCPAP cell line (Fig. 4A, B).

### Correlations between cell polarity, cell speed and ZEB1 non-coding RNA expression are lowered by CD73 loss

**Fig. 5:**
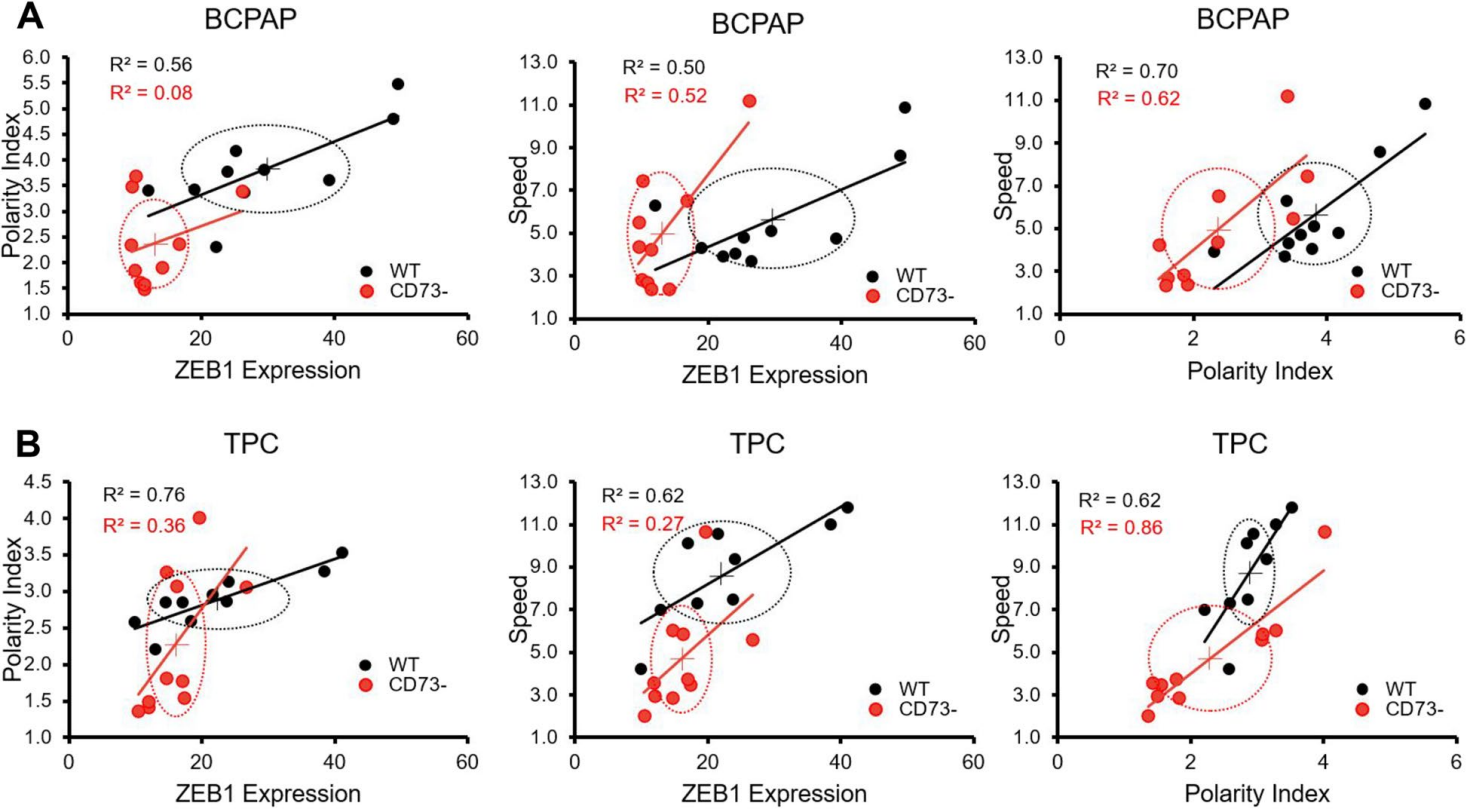
ZEB1 reporter expression correlation with cell migration speed (μm/h) and polarity index. **A** Correlations among cell migration speed, polarity index, and ZEB1 reporter expression in BCPAP WT and BCPAP CD73- cell lines. **B** Correlations among cell migration speed, polarity index, and ZEB1 reporter expression in TPC WT and TPC CD73- cell lines. Ellipses represent standard deviations around the means (crosses)

When assessing the correlations of cell migration parameters, specifically, cell polarity index, cell speed, and ZEB1 reporter expression between WT and CD73- cells, it became evident that WT cells exhibited positive correlation coefficients. However, in some cases, the correlation was lost in CD73- cells, in both TPC and BCPAP cell lines (Fig. 5A, B). The coefficients between cell migration speed and polarity index remained consistent regardless of cell editing. Furthermore, ZEB1 reporter expression and polarity index quantifications were notably higher in BCPAP WT compared to BCPAP CD73- cells. Conversely, for TPC WT and TPC CD73- cells, this difference was observed primarily in cell migration speed quantification.

### Partial-EMT is associated with BRAF mutations and ZEB1/CD73 expression

**Fig. 6:**
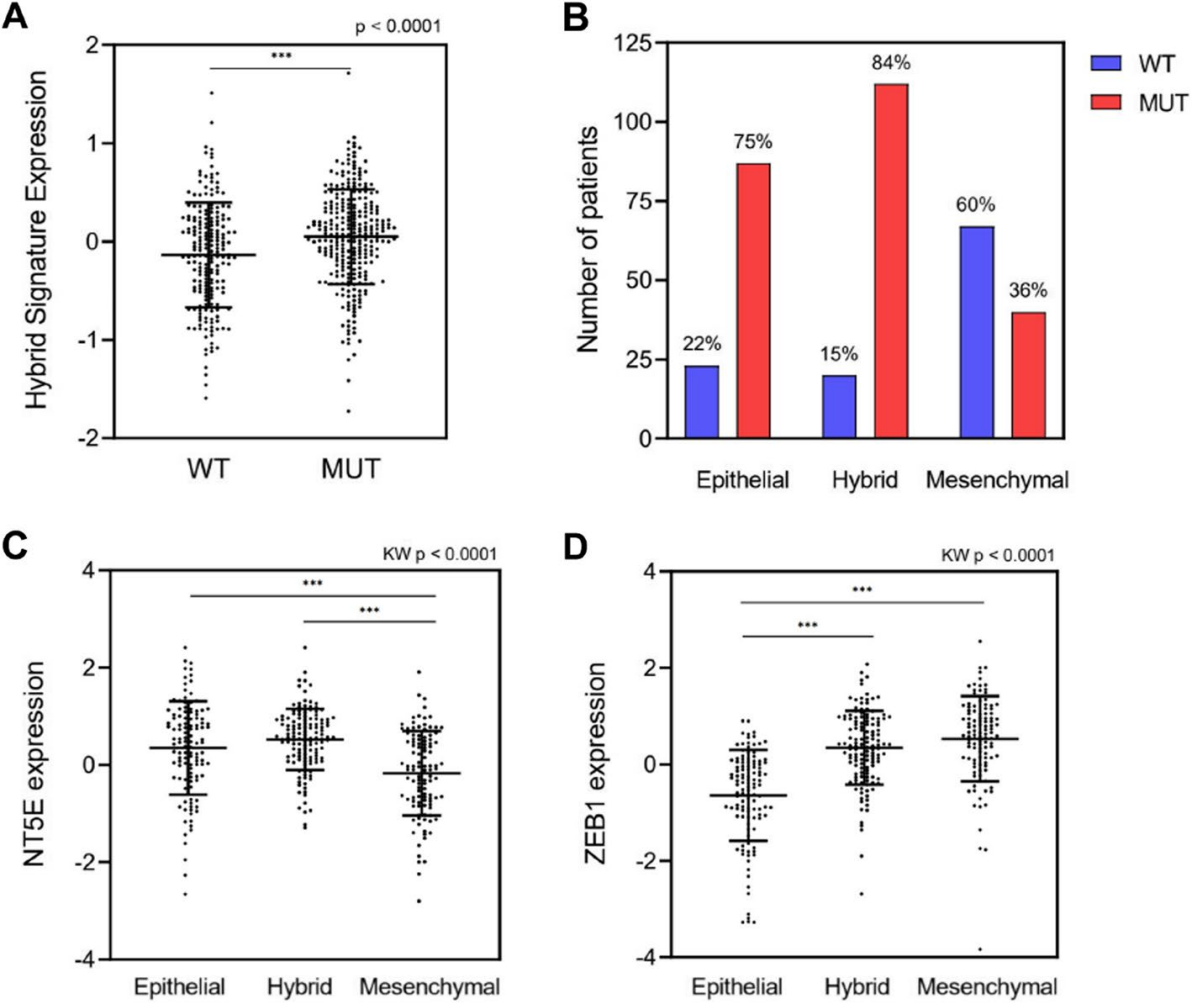
Association between EMT transition states, BRAF mutations, and *ZEB1* and *NT5E* expressions. **A** Expression of a partial-EMT (hybrid state) signature in PTC samples from a TCGA cohort divided into wild type (WT, *n* = 201) and BRAF mutated (MUT, *n* = 286). **B** Classification of samples according to EMT profile (Epithelial: *n* = 115, Hybrid: *n* = 133, or Mesenchymal: *n* = 110). The number and percentage of patients in each EMT state group with wild type or BRAF mutated status are presented. **C** *NT5E* expression levels across EMT state groups. **D** *ZEB1* expression levels across EMT state groups. *P* values (p) are indicated. Horizontal lines represent the median and first and third quartiles. **** = *p* ≤ 0.0001

To discern EMT states in PTC and explore their association with BRAF mutations and CD73 and ZEB1 expressions, we performed an analysis of RNAseq data derived from PTC samples within a TCGA cohort. Initially, we observed that *NT5E* (CD73) expression was elevated in samples from more aggressive PTC subtypes, as well as in those with BRAF mutations (Supplementary Figs. 1A, B). We further evaluated the association between BRAF mutations and EMT transition states using gene signatures for each state, including epithelial, hybrid, and mesenchymal. Notably, the expression of the hybrid state (partial-EMT) signature exhibited a significant increase in BRAF-mutated samples (Fig. 6A). Additionally, expressions of epithelial and mesenchymal signatures were utilized to classify patients according to their EMT profile (Supplementary Figs. 1C, D). The EMT hybrid state group mainly consisted of BRAF-mutated samples (84%), which also constituted the majority of samples in the epithelial state but the minority in the mesenchymal state (Fig. 6B). The expression of *NT5E* exhibited a significant increase in both epithelial and hybrid states compared to the mesenchymal state, whereas *ZEB1* expression was higher in the mesenchymal and hybrid states (Fig. 6C, D). In univariate analysis, EMT states did not exhibit associations with age ≥ 55 years old (*p* = 0.075), lymph node metastases (*p* = 0.225), or gender (*p* = 0.102) (Supplementary Table 1). Conversely, EMT states were significantly associated with stages I/II vs III/IVA/IVC AJCC of the 7th edition of the American Joint Committee on Cancer (*p* = 0.005), histological classification (*p* < 0.0001), extrathyroidal extension (*p* = 0.008) and BRAF mutation (*p* < 0.0001). Upon adjusting for these significant variables in a multivariable model, only the presence of BRAF mutation remained independently associated with EMT states (Supplementary Table 1).

## Discussion

This study utilized the editing of PTC cell lines to silence CD73 and express the ZEB1 reporter. This approach facilitated the exploration of potential regulatory mechanisms linking the EMT and the adenosinergic pathway. Notably, a prior study has established a connection between CD73 mRNA levels and clinical characteristics of patients with PTC, including metastatic lymph nodes and larger tumor size [11]. Similarly, ZEB1 expression is associated with clinicopathological features such as advanced tumor-node metastasis stage, lymph node metastasis, and distant metastasis in patients with thyroid cancer [24].

ZEB1 has been implicated in acting in the invasive front of tumors, not only through its induction in cancer cells of a motile dedifferentiated phenotype, but also via differential regulation of genes involved in stromal remodeling [25]. Furthermore, prior investigations have demonstrated the co-enrichment of CD73 and ZEB1 gene expressions in lung cancer cell lines and tumors [26]. Interestingly, previous studies have shown an elevation in CD73 expression in sections of PTC compared to follicular epithelial cells of a normal thyroid. This heightened expression was particularly pronounced at the invasive front, suggesting a potential correlation between ZEB1 and CD73 in the context of tumor invasiveness [10, 12].

To assess the significance of CD73 in regulating ZEB1 expression in PTC, we examined the regulation of ZEB1 through GFP fluorescence controlled by the 3’UTR of ZEB1 in CD73-negative cell lines. Our findings revealed a significant reduction in ZEB1 reporter expression in BCPAP CD73- cells compared to their WT counterparts. Despite indications of CD73 influencing the expression of the mesenchymal marker in various methodologies applied to BCPAP, this effect was not consistently observed in TPC cells. The disparity in results across cell lines underscores the complexity and context-dependency of CD73 impact on ZEB1, warranting further investigation and analysis.

When assessing the cell polarity index, we observed that BCPAP CD73- cells displayed significantly lower polarity compared to BCPAP WT cells. This implies the significance of CD73 in promoting a migratory phenotype, consistent with a previous study involving CD73-siRNA transfected cells, which established a connection between CD73 to a reduction in both migration and invasion processes [9]. The migration analysis using the scratch wound assay revealed a significant difference in the gap size between BCPAP WT cells and BCPAP CD73- cells, although this difference was not observed in TPC.

Due to the intricate and context-specific nature of gene regulation linked to ZEB1 expression, additional factors may contribute to variations in results between BCPAP and TPC cell lines. BCPAP harbors BRAF^V600E^ mutation, which is associated with more aggressive clinicopathologic characteristics, and exhibits a heightened expression of genes associated with cell cycle and DNA replication [27]. Whereas TPC is characterized by the RET/PTC1 rearrangement, which is associated with a relatively benign clinical course [28–32].

For the BCPAP WT and TPC WT cell lines, there was a moderate correlation between ZEB1 reporter expression, cell polarity index, and cell speed. However, in the case of CD73- cells, the correlations between these parameters were low to negligible. This suggests that CD73 silencing loosened the link between non-coding RNA regulation of ZEB1 and cell polarity index, as well as cell migration speed. Previous studies have indicated that CD73 can promote cell migration in various types of cancer since it not only serves as an enzyme but also as an adhesion molecule that facilitates the migration of both normal and malignant cells [33]. As expected, a strong correlation was observed between cell speed and polarity in both cell lines, regardless of their CD73 status, as effective cell translocation necessitates cell polarity [34]. Further investigation is warranted to elucidate the potential mechanisms through which CD73 and ZEB1 may modulate these morphological and functional cellular changes, as these mechanisms remain incompletely understood.

The analysis of RNA-seq data from PTC samples revealed a prominent occurrence of the hybrid phenotype specifically in samples harboring BRAF mutations. This observation aligns with previous research indicating that the network of EMT transcription factors is significantly restructured in response to NRAS/BRAF activation, leading to the upregulation of TWIST1 and ZEB1 [35]. An emerging approach for assessing mutation associations in PTC cell lines involves exploring the epigenetic landscape. Recently, it was demonstrated that the hypomethylation of the cg23172664 site was associated with a BRAF-like phenotype and lower levels of *NT5E* expression in PTC [36]. Overall, additional exploration is needed to assess potential associations among specific tumor mutations, such as BRAF, EMT, and adenosinergic pathway.

Notably, the expression of *NT5E* was found to be heightened in the more aggressive subtypes of PTC, aligning with previously reported data [11]. Its expression was also higher in epithelial and hybrid states compared to the mesenchymal state, while *ZEB1* expression was more pronounced in hybrid and mesenchymal states compared to the epithelial state. *ZEB1* is present in early hybrid, late hybrid, and full EMT states [22], supporting our findings. However, *NT5E* expression was unexpectedly lower in the mesenchymal state, highlighting the necessity for additional research.

The roles of CD73 in cancer are multifaceted, acting as both pro-tumor and anti-tumor agents in a tumor-specific manner [37]. Most importantly, both *ZEB1* and *NT5E* are highly expressed in the hybrid state, suggesting that the EMT state may be relevant for the association between *ZEB1* and *NT5E*. In this context, their prevalence in hybrid states aligns with a previous suggestion that thyroid cancer progression is characterized by an incomplete EMT [38]. Remarkably, recent research has introduced RGD-conjugated chitosan lactate nanoparticles encapsulating CD73 and ZEB1 siRNA molecules. Administration of these nanoparticles was associated with a significant reduction in migration and proliferation in vitro, as well as tumor suppression and prolonged survival in vivo [39].

In conclusion, our study highlights the significance of targeting CD73 in cancer beyond its role in the immune system and emphasizes the importance of understanding its interactions with EMT markers, such as ZEB1. The interplay between EMT and the adenosinergic pathway remains a relatively unexplored area, especially in the context of PTC. Hence, our findings offer valuable insights that may serve as a foundation for further investigations in this field, potentially contributing to advancements in cancer therapeutics in the future.

## Conclusion

Our research demonstrates the involvement of CD73 in modulating the non-coding RNA regulation of ZEB1 through its 3’UTR in cell lines derived from PTC. The suppression of CD73 results in a decrease in ZEB1 levels and modifies its correlations with cell polarity and migration speed. By shedding light on the interactions between these pivotal elements of EMT and adenosinergic signaling, we aim to contribute to the development of innovative approaches for treating PTC and potentially other cancers.

### Supplementary Information

**Additional file 1: Figure S1.** *NT5E* expression across PTC samples and definition of patient groups based on EMT profile. (A) *NT5E* expression across PTC samples from follicular, classical, and tall cell subtypes. (B) *NT5E* expression in wild type and BRAF mutated PTC samples. (C) Pearson correlation between the expressions of a mesenchymal and an epithelial signature. The dotted lines represent the cut-off points that divide the patients into 4 groups based on their expressions: one in the correlation line and a second perpendicular to that line. Pearson’s correlation coefficient (r) and *p*-value (p) are indicated. (D) Expression of a partial-EMT (hybrid state) signature across the defined groups was used to validate the patient separation. * = *p* ≤ 0.05; **** = *p* ≤ 0.0001.

**Additional file 2: Table S1.** Clinicopathological characteristics associated with EMT states in Papillary Thyroid Carcinoma.

## Data Availability

The data supporting these findings are available from the corresponding author upon reasonable request.

## References

[1] Brabletz S, Schuhwerk H, Brabletz T, Stemmler MP (2021). Dynamic EMT: a multi-tool for tumor progression. EMBO J.

[2] Iser IC, Pereira MB, Lenz G, Wink MR (2017). The epithelial-to-mesenchymal transition-like process in glioblastoma: an updated systematic review and in silico investigation. Med Res Rev.

[3] Brabletz T, Kalluri R, Nieto MA, Weinberg RA (2018). EMT in cancer. Nat Rev Cancer.

[4] Lu J, Fei F, Wu C, Mei J, Xu J, Lu P (2022). ZEB1: catalyst of immune escape during tumor metastasis. Biomed Pharmacother.

[5] Guo Y, Lu X, Chen Y, Rendon B, Mitchell RA, Cuatrecasas M, Cortés M, Postigo A, Liu Y, Dean DC. Zeb1 induces immune checkpoints to form an immunosuppressive envelope around invading cancer cells. Sci Adv. 2021; 10.1126/sciadv.abd7455. 10.1126/sciadv.abd7455 PMC8139582 34020945

[6] Perez-Oquendo M, Gibbons DL. Regulation of ZEB1 function and molecular associations in tumor progression and metastasis. Cancers. 2022; 10.3390/cancers14081864. 10.3390/cancers14081864 PMC9031734 35454770

[7] Humphries B, Yang C (2015). The microRNA-200 family: small molecules with novel roles in cancer development, progression and therapy. Oncotarget.

[8] Di Virgilio F, Adinolfi E (2017). Extracellular purines, purinergic receptors and tumor growth. Oncogene.

[9] Jeong YM, Cho H, Kim T-M, Kim Y, Jeon S, Bychkov A, Jung CK. CD73 overexpression promotes progression and recurrence of papillary thyroid carcinoma. Cancers. 2020; 10.3390/cancers12103042. 10.3390/cancers12103042 PMC7603384 33086655

[10] Kondo T, Nakazawa T, Murata S-I, Katoh R (2006). Expression of CD73 and its ecto-5′-nucleotidase activity are elevated in papillary thyroid carcinomas. Histopathology.

[11] Bertoni APS, Bracco PA, de Campos RP, Lutz BS, Assis-Brasil BM, de Meyer EL S, Saffi J, Braganhol E, Furlanetto TW, Wink MR (2019). Activity of ecto-5′-nucleotidase (NT5E/CD73) is increased in papillary thyroid carcinoma and its expression is associated with metastatic lymph nodes. Mol Cell Endocrinol.

[12] Monteiro I, Missiaglia E, Sciarra A, Santos JV, Bouilly J, Romero P, Sempoux C, de Leval L (2021). CD73 expression in normal, hyperplastic, and neoplastic thyroid: a systematic evaluation revealing CD73 overexpression as a feature of papillary carcinomas. Virchows Arch.

[13] Bertoni APS, de Campos RP, Tsao M, Braganhol E, Furlanetto TW, Wink MR (2018). Extracellular ATP is differentially metabolized on papillary thyroid carcinoma cells surface in comparison to Normal cells. Cancer Microenviron.

[14] Gharib H, Papini E, Garber JR, Duick DS, Harrell RM, Hegedüs L, Paschke R, Valcavi R, Vitti P, AACE/ACE/AME Task Force on Thyroid Nodules (2016). American association of clinical endocrinologists, American college of endocrinology, and Associazione medici endocrinologi medical guidelines for clinical practice for the diagnosis and management of thyroid nodules--2016 update. Endocr Pract.

[15] Haugen BR, Alexander EK, Bible KC (2016). 2015 American Thyroid Association Management Guidelines for adult patients with thyroid Nodules and differentiated thyroid Cancer: the American Thyroid Association Guidelines task force on thyroid Nodules and differentiated thyroid Cancer. Thyroid.

[16] Iser IC, Vedovatto S, Oliveira FD, Beckenkamp LR, Lenz G, Wink MR (2022). The crossroads of adenosinergic pathway and epithelial-mesenchymal plasticity in cancer. Semin Cancer Biol.

[17] Allard B, Turcotte M, Stagg J (2012). CD73-generated adenosine: orchestrating the tumor-stroma interplay to promote cancer growth. J Biomed Biotechnol.

[18] Ran FA, Hsu PD, Wright J, Agarwala V, Scott DA, Zhang F (2013). Genome engineering using the CRISPR-Cas9 system. Nat Protoc.

[19] Reinhardt J, Landsberg J, Schmid-Burgk JL (2017). MAPK signaling and inflammation link melanoma phenotype switching to induction of CD73 during immunotherapy. Cancer Res.

[20] Cerami E, Gao J, Dogrusoz U (2012). The cBio cancer genomics portal: an open platform for exploring multidimensional cancer genomics data. Cancer Discov.

[21] Gao J, Aksoy BA, Dogrusoz U (2013). Integrative analysis of complex cancer genomics and clinical profiles using the cBioPortal. Sci Signal.

[22] Pastushenko I, Blanpain C (2019). EMT transition states during tumor progression and metastasis. Trends Cell Biol.

[23] Pereira MB, Barros LRC, Bracco PA, Vigo A, Boroni M, Bonamino MH, Lenz G (2018). Transcriptional characterization of immunological infiltrates and their relation with glioblastoma patients overall survival. Oncoimmunology.

[24] Zhang Y, Liu G, Wu S, Jiang F, Xie J, Wang Y (2016). Zinc finger E-box-binding homeobox 1: its clinical significance and functional role in human thyroid cancer. Onco Targets Ther.

[25] Sánchez-Tilló E, de Barrios O, Siles L, Amendola PG, Darling DS, Cuatrecasas M, Castells A, Postigo A (2013). ZEB1 promotes invasiveness of colorectal carcinoma cells through the opposing regulation of uPA and PAI-1. Clin Cancer Res.

[26] Jin R, Liu L, Xing Y (2020). Dual mechanisms of novel CD73-targeted antibody and antibody-drug conjugate in inhibiting lung tumor growth and promoting antitumor immune-effector function. Mol Cancer Ther.

[27] Saiselet M, Floor S, Tarabichi M, Dom G, Hébrant A, van Staveren WCG, Maenhaut C (2012). Thyroid cancer cell lines: an overview. Front Endocrinol.

[28] Chakraborty A, Narkar A, Mukhopadhyaya R, Kane S, D’Cruz A, Rajan MGR (2012). BRAF V600E mutation in papillary thyroid carcinoma: significant association with node metastases and extra thyroidal invasion. Endocr Pathol.

[29] Chen X, Lin S, Lin Y, Wu S, Zhuo M, Zhang A, Zheng J, You Z (2022). BRAF-activated WT1 contributes to cancer growth and regulates autophagy and apoptosis in papillary thyroid carcinoma. J Transl Med.

[30] Tang K-T, Lee C-H (2010). BRAF mutation in papillary thyroid carcinoma: pathogenic role and clinical implications. J Chin Med Assoc.

[31] Nikiforov YE (2002). RET/PTC rearrangement in thyroid tumors. Endocr Pathol.

[32] Pilli T, Prasad KV, Jayarama S, Pacini F, Prabhakar BS (2009). Potential utility and limitations of thyroid cancer cell lines as models for studying thyroid cancer. Thyroid.

[33] Hu X-M, Shi N-R, Zhang J-Z, Zuo Y-Q, Wang X, Zhao Y-F, Wu J-S (2023). CD73: friend or foe in lung injury. Int J Mol Sci.

[34] Libotte T, Kaiser HW, Alt W, Bretschneider T (2001). Polarity, protrusion-retraction dynamics and their interplay during keratinocyte cell migration. Exp Cell Res.

[35] Caramel J, Papadogeorgakis E, Hill L (2013). A switch in the expression of embryonic EMT-inducers drives the development of malignant melanoma. Cancer Cell.

[36] Bertoni APS, Valandro CF, Brasil RÁ, Zeiser FA, Wink MR, Furlanetto TW, da Costa CA (2023). NT5E DNA methylation in papillary thyroid cancer: novel opportunities for precision oncology. Mol Cell Endocrinol.

[37] Alcedo KP, Bowser JL, Snider NT (2021). The elegant complexity of mammalian ecto-5′-nucleotidase (CD73). Trends Cell Biol.

[38] Baldini E, Tuccilli C, Pironi D, et al. Expression and clinical utility of transcription factors involved in epithelial-mesenchymal transition during thyroid Cancer progression. J Clin Med Res. 2021; 10.3390/jcm10184076. 10.3390/jcm10184076 PMC8469282 34575184

[39] Alzamely KO, Hajizadeh F, Heydari M (2021). Combined inhibition of CD73 and ZEB1 by Arg-Gly-asp (RGD)-targeted nanoparticles inhibits tumor growth. Colloids Surf B: Biointerfaces.

