# Supplementary figures and images for: CD73 mitigates ZEB1 expression in papillary thyroid carcinoma
Source: Cell Commun Signal. 2024 Feb 22;22:145. doi: 10.1186/s12964-024-01522-z (PMC10882796; doi:10.1186/s12964-024-01522-z)

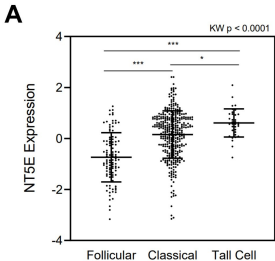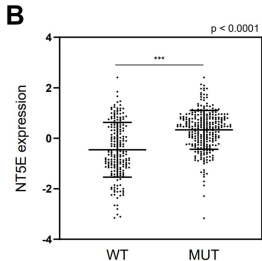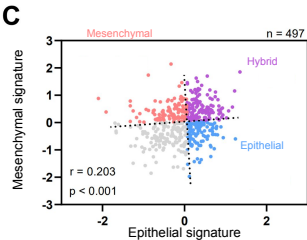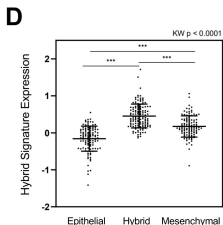

Supplement: Supplementary file 1 — Additional file 1: Figure S1. NT5E expression across PTC samples and definition of patient groups based on EMT profile. (A) NT5E expression across PTC samples from follicular, classical, and tall cell subtypes. (B) NT5E expression in wild type and BRAF mutated PTC samples. (C) Pearson correlation between the expressions of a mesenchymal and an epithelial signature. The dotted lines represent the cut-off points that divide the patients into 4 groups based on their expressions: one in the correlation line and a second perpendicular to that line. Pearson’s correlation coefficient (r) and p-value (p) are indicated. (D) Expression of a partial-EMT (hybrid state) signature across the defined groups was used to validate the patient separation. * = p ≤ 0.05; **** = p ≤ 0.0001. [file 12964_2024_1522_MOESM1_ESM.pdf]
