# Supplementary material for: CD73 mitigates ZEB1 expression in papillary thyroid carcinoma
Source: Cell Commun Signal. 2024 Feb 22;22:145. doi: 10.1186/s12964-024-01522-z (PMC10882796; doi:10.1186/s12964-024-01522-z)
Supplement: Supplementary file 2 — Additional file 2: Table S1. Clinicopathological characteristics associated with EMT states in Papillary Thyroid Carcinoma. [file 12964_2024_1522_MOESM2_ESM.pdf]

Feature	*N* (%)	EMT states - Univariate analysis				EMT states - Multivariate analysis			
		Epithelial	Hybrid	Mesenchymal	*P* value	B	SE	beta	*P* value
Classification									
Classical	265	94 (35.5)	104 (39.2)	67 (25.3)	<0.0001	-.007	.0263	.069	.261
Follicular	39	5 (12.8)	6 (15.4)	28 (71.8)					
Tall Cell	32	9 (28.1)	16 (50.0)	7 (21.9)					
Extrathyroidal extension									
No	184	75 (40.8)	64 (34.8)	45 (24.5)	0.008	-.064	.0554	1.318	.251
Yes	110	27 (24.5)	56 (51.0)	27 (24.5)					
Metastatic lymph nodes									
No	167	51 (30.5)	71 (42.5)	45 (26.9)	0.225				
Yes	173	59 (34.1)	58 (33.5)	56 (32.4)					
Gender									
Female	248	88 (35.5)	92 (37.1)	68 (27.4)	0.102	-.067	0.533	1.558	.212
Male	92	22 (23.9)	37 (40.2)	33 (35.9)					
Age (years)									
< 55	234	87 (35.8)	90 (37.0)	66 (27.2)	0.075				
≥ 55	97	23 (23.7)	39 (40.2)	35 (36.1)					
BRAF									
No	104	28 (26.9)	34 (32.7)	42 (40.4)	<0.0001	-0,170	.0493	11.892	<0.0001
Yes	189	72 (38.1)	86 (45.5)	31 (16.4)					
AJCC stages									
I and II	201	79 (39.3)	67 (33.3)	55 (27.4)	0.005	NA			
III and IV	138	31 (22.5)	61 (44.2)	46 (33.33)					

Data are given as number (n) and percentage (%) or mean and 95% confidence limits. AJCC: The American Joint Committee on Cancer7th edition. NA: not included for colinearity. SE: standard error.
